# Unexpectedly High Prevalence of Cytomegalovirus DNAemia in Older Children and Adolescents With Perinatally Acquired Human Immunodeficiency Virus Infection

**DOI:** 10.1093/cid/ciy961

**Published:** 2019-03-04

**Authors:** Louis-Marie Yindom, Victoria Simms, Edith D Majonga, Grace McHugh, Ethel Dauya, Tsitsi Bandason, Helene Vincon, Jamie Rylance, Shungu Munyati, Rashida A Ferrand, Sarah L Rowland-Jones

**Affiliations:** 1 University of Oxford, Nuffield Department of Medicine, United Kingdom; 2 Department of Clinical Research, London School of Hygiene and Tropical Medicine, United Kingdom; 3 Biomedical Research and Training Institute, Harare, Zimbabwe; 4 Department of Clinical Research, Liverpool School of Tropical Medicine, United Kingdom

**Keywords:** CMV, HIV, adolescent, chronic lung disease, stunting

## Abstract

**Background:**

Older children and adolescents with perinatally acquired human immunodeficiency virus (PHIV) infection in Africa experience multiple comorbidities that are not typical of HIV-associated opportunistic infections, including growth impairment and chronic lung disease. We examined associations between plasma cytomegalovirus (CMV) DNA and lung function and growth.

**Methods:**

Plasma CMV DNA loads were measured children aged 6–16 years with PHIV (n = 402) and HIV-uninfected controls (n = 224). The HIV-infected children were either newly diagnosed or known HIV infected and stable on antiretroviral therapy (ART) for >6 months. CMV DNA loads were measured using quantitative polymerase chain reaction. CMV DNAemia was modeled as a time-varying outcome using longitudinal mixed-effects logistic regression.

**Results:**

At enrollment, CMV DNAemia ≥1000 copies/mL (defined as “clinically significant”) was detected in 5.8% of uninfected children, 14.7% of HIV-infected participants stable on ART, and 22.6% of HIV-infected ART-naive children (χ^2^ = 23.8, *P* < .001). The prevalence of CMV DNAemia ≥1000 copies/mL was associated with CD4 counts <350 cells/µL. Among HIV-infected ART-naive children, the presence of CMV DNAemia of ≥1000 copies/mL was independently associated with reduced lung function (adjusted odds ratio [aOR] = 3.23; 95% confidence interval [CI], 1.23–8.46; *P* = .017). Among ART-treated children, stunting was associated with CMV DNAemia of ≥1000 copies/mL (aOR = 2.79; 95% CI, 0.97–8.02; *P* = .057).

**Conclusions:**

Clinically significant levels of CMV DNAemia were common in older children with PHIV, even those on ART, suggesting a role for inadequately controlled CMV infection in the pathogenesis of PHIV comorbidities in Africa.

## Teaser

Cytomegalovirus (CMV) is ubiquitous in Africa, infecting all children by age 18 months. We report that clinically significant levels of CMV DNAemia in older children with perinatally acquired human immunodeficiency virus infection (PHIV) associate with 2 serious long-standing PHIV comorbidities, stunting and chronic lung disease.

Before the widespread availability of antiretroviral therapy (ART), pediatric human immunodeficiency virus (HIV) in Africa was characterized by rapid disease progression, with <50% of children with perinatally acquired HIV infection (PHIV) surviving beyond 2 years [1]. At that time, the survival of HIV-infected children into adolescence seemed unlikely. However, it is now thought that one third of children with PHIV progress more slowly and can survive into their teens, even without ART [2]. The ART scale-up for PHIV in Africa has led to dramatic falls in mortality, leading to increasing numbers of ART-treated children reaching adolescence [3]. Long-term survival with PHIV, even for those on ART, is associated with significant health problems that are not typical of HIV-associated opportunistic infections or AIDS-defining illnesses [4]. Reports from sub-Saharan Africa have described significant morbidity in older children with PHIV [4], including chronic lung disease (CLD), cardiac abnormalities [5], growth retardation, pubertal delay, and neurocognitive disorders. Many of these PHIV comorbidities have not previously been reported outside Africa, and their pathogenesis is poorly understood.

The most serious comorbidity described in older children with PHIV is CLD, reported in more than 30% of HIV-infected children aged >10 years in HIV care but frequently misdiagnosed and treated as pulmonary tuberculosis [6]. The severity of CLD was only recently recognized, and little is known about its etiology [6, 7]. Lung function and radiological abnormalities in older children with PHIV show no associations with either treatment status or ART duration, suggesting that once established, this form of CLD does not respond to ART. Indeed, a recent study showed that 25% of older children with PHIV on ART (median treatment duration, 4.7 years; viral suppression below detection in 79%) reported symptoms of CLD, predominantly cough and dyspnea [8].

We hypothesized that cytomegalovirus (CMV) might contribute to the poor health of older children with PHIV, in view of the distinct epidemiology of CMV infection in sub-Saharan Africa. Most African infants acquire CMV infection in the first year of life, in contrast to developed countries where CMV infection occurs significantly later (mean age of 28.6 years in 1 large US study [9]). In the Gambia, 85% of infants acquired CMV infection by 1 year of age, increasing to 100% by 18 months [10]. HIV-uninfected infants generate potent cellular responses to CMV that are associated with viral control, although urinary CMV excretion may continue intermittently to age 5 years [11]. It is therefore highly likely that most African children with PHIV will acquire CMV coinfection in infancy.

CMV coinfection in ART-untreated infants with PHIV can lead to rapid disease progression [12, 13] and often infects the lungs, causing pneumonitis [14]. No studies have examined the long-term impact of infant CMV pneumonitis on subsequent lung function. However, it is plausible that repeated episodes of CMV pneumonitis or inflammation driven by subclinical CMV replication in HIV-infected children could lead to CLD.

We hypothesized that ART-untreated infants with PHIV who acquire CMV coinfection in early life fail to establish adequate immune control of CMV, leading to episodes of CMV reactivation and/or persistent CMV replication that may increase immune activation and drive some of the comorbidities described in adolescent survivors of PHIV.

## METHODS

### Study Setting and Participants

We studied 626 participants within 2 prospective cohort studies investigating the natural history of comorbidities that affect children with PHIV, including lung function and growth. The ZENITH cohort recruited children aged 6–16 years who were newly diagnosed with HIV following provider-initiated HIV testing in primary healthcare clinics in Harare, Zimbabwe [15]. The INHALE cohort recruited children in the same age group who had been taking ART for >6 months from an HIV clinic at Harare Children’s Hospital, which served the same catchment population as the primary healthcare clinics. Inclusion/exclusion criteria are described elsewhere [16, 17]. PHIV diagnosis was made using criteria previously established in Zimbabwe [4].

Spirometry was performed at baseline using EasyOne World spirometers (ndd Medical Technologies, Zurich, Switzerland) according to American Thoracic Society standard procedures [7]. The highest forced expiratory volume in the first second of expiration (FEV1) and forced vital capacity (FVC) measurements, that is, the volume of air exhaled with maximum effort after a full inspiration, were used, with other indices recorded from the best trace (largest total of FEV1 and FVC). Using the Global Lung Function Initiative 2012 reference ranges (accounting for height, sex, age, and ethnicity), lung function was considered abnormal if either FVC or FEV1:FVC ratio *z* scores were less than −1.64 (below the 10th centile). Height was recorded at baseline for all participants, and HIV-infected participants were followed for 18 months, with clinical and laboratory examinations every 3 months. Blood samples were collected and plasma was separated on the same day at the Biomedical Research and Training Institute (BRTI) in Harare, then stored at −80°C until required.

**Figure 1.**
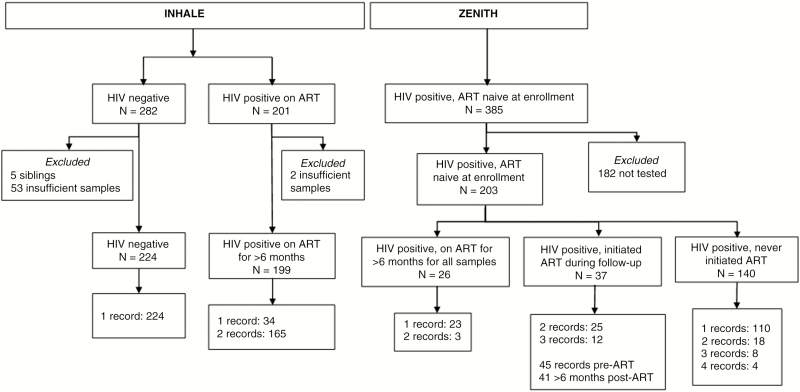
Flow chart of study participants. Abbreviations: ART, antiretroviral therapy; HIV, human immunodeficiency virus; INHALE, INvestigation of Heart And Lung disEase among older children in Harare; ZENITH, Zimbabwe study for ENhancing Testing and Improving Treatment of HIV in children.

Children who tested HIV negative in the same age group as cohort participants joined the study as population controls and provided a single blood sample without further follow-up. Recruitment started in January 2013, and follow-up lasted until April 2015 (Figure 1).

### Extraction of CMV DNA From Plasma

Total viral nucleic acids were extracted at the BRTI laboratories in Harare from 200 µL plasma using the QIAamp MinElute Virus Spin Kit (Qiagen, Hilden, Germany). Then, 60 µL of total viral nucleic acids including CMV DNA were eluted and immediately stored at −80°C for subsequent testing.

### CMV Detection and Quantification

CMV DNAemia detection and quantification was performed by quantitative polymerase chain reaction (PCR) using the RealStar CMV PCR kit v1.0 (Altona Diagnostics, Hamburg, Germany). CMV DNA was quantified with the QuantStudio 3 Real-Time PCR System (Applied Biosystems, CA) (see Supplementary Methods for details).

### Statistical Analyses

“Clinically relevant” CMV viral load (VL) was defined as plasma CMV VL ≥1000 copies/mL. Stunting was defined as a height-for-age more than 2 standard deviations below the mean, using World Health Organization reference standards. χ^2^ tests were used for univariate analysis. For multivariate analysis of time-varying outcomes, we used longitudinal mixed-methods logistic regression. ART-naive participants and those stable on ART were modeled separately. Data were analyzed using Stata v14.1 (StataCorp, College Station, TX). As a sensitivity analysis, we also examined associations of risk factors with prevalence of any detectable CMV DNA (see Supplementary Methods for details).

### Ethical Considerations

Informed consent (parent or guardian) and assent (participant) were obtained from all participants prior to enrollment. The Medical Research Council of Zimbabwe, the BRTI Institutional Review Board, and the Ethics Committees of Harare City Health Department, Harare Central Hospital, and the London School of Hygiene and Tropical Medicine approved the study.

## RESULTS

### Baseline Characteristics of Study Participants

A total of 626 children and adolescents age 6–16 years were included. When the first sample was taken, 177 were HIV infected and ART naive, 225 were HIV infected on ART for >6 months, and 224 were uninfected controls (Figure 1). Baseline characteristics are provided in Table 1. During follow-up, 37 of the 177 originally ART-naive participants initiated ART and had a sample taken at least 6 months after starting therapy (Figure 1). Their pre-ART samples were included in the ART-naive analysis, and post-ART samples were included in the analysis of ART-experienced participants. There were 889 observations: 626 at baseline, 33 at 6 months, 37 at 12 months, 159 at 18 months, and 34 at 24 months. The median age at recruitment was similar between groups (11 years), and 50% were female. Stunting was more prevalent among HIV-positive children than their uninfected counterparts (32.5% vs 9.6%). Stunting was also associated with age; prevalence was 13.0% for children aged 6–9 years, 23.3% for children aged 10–12 years, and 39.6% for children aged >13 years (χ^2^ = 37.5, *P* < .001). Abnormal lung function (defined as described above) was common, ranging from 15.5% in the uninfected group to 24.1% in the group stable on ART and 29.4% in the ART-naive HIV-positive group.

**Table 1.** Baseline Characteristics of the Study Population and Prevalence of High Cytomegalovirus DNAemia

Characteristic	Category	HIV Negative^a^		HIV Positive on ART^a^		HIV Positive ART-Naive^a^	
		N (%)	CMV ≥1000 n/N (%)	N (%)	CMV ≥1000 n/N (%)	N (%)	CMV ≥1000 n/N (%)
Gender	Male	108 (48.2)	5/108 (4.6)	114 (50.7)	17/114 (14.9)	90 (50.9)	19/90 (21.1)
	Female	116 (51.8)	8/116 (6.9)	111 (49.3)	16/111 (14.4)	87 (49.1)	21/87 (24.1)
Age (years)	6–9	83 (37.1)	9/83 (10.8)	72 (32.0)	10/72 (13.9)	73 (41.2)	16/73 (23.3)
	10–12	72 (32.1)	3/72 (4.2)	92 (40.9)	13/92 (14.1)	58 (32.8)	11/58 (19.0)
	13–16	69 (30.8)	1/69 (1.5)	61 (27.1)	10/61 (16.4)	46 (26.0)	12/46 (26.1)
Height for age^b^	Stunted	21 (9.6)	1/21 (4.8)	77 (34.3)	12/77 (15.6)	49 (29.2)	20/49 (40.8)
	Not stunted	198 (90.4)	12/198 (6.1)	147 (65.7)	21/147 (14.3)	119 (70.8)	17/119 (14.3)
Lung function^c^	Normal	158 (84.5)	10/158 (6.3)	148 (75.9)	23/148 (15.5)	108 (63.3)	22/108 (20.4)
	Reduced forced vital capacity	22 (11.8)	1/22 (4.6)	38 (19.5)	4/38 (10.5)	29 (17.5)	9/29 (31.0)
	Obstruction	7 (3.8)	0 (0.0)	9 (4.6)	1/9 (11.1)	16 (9.6)	5/16 (31.3)
CD4 count (cells/µL)^d^	≤349	…		23 (10.5)	6/23 (26.1)	48 (35.1)	11/48 (22.9)
	350–499	…		39 (17.7)	5/39 (12.8)	22 (14.9)	3/22 (13.6)
	≥500	…		158 (71.8)	20/158 (12.7)	67 (50.0)	13/67 (19.4)
CMV VL (copies/mL of plasma)	Undetectable	204 (91.1)		171 (76.0)		109 (61.6)	
	1–999	7 (3.1)		21 (9.3)		28 (15.8)	
	1000–9999	13 (5.8)		27 (12.0)		34 (19.2)	
	≥10000	0 (0.0)		6 (2.7)		6 (3.4)	
HIV RNA (copies/mL of plasma)	≤999	…	…	180 (84.1)	25/178 (14.0)	…	…
	1000–9999	…	…	6 (2.8)	0/6 (0.0)	…	…
	≥10000	…	…	28 (13.1)	5/28 (17.9)	…	…

Abbreviations: ART, antiretroviral therapy; CMV, cytomegalovirus; HIV, human immunodeficiency virus; VL, viral load.

^a^Of the total number of participants, 224 were HIV negative, 225 HIV positive on ART, and 177 HIV positive/ART-naive.

^b^Data on stunting were missing for 5 HIV-uninfected and 9 HIV-positive/ART-naive participants.

^c^The numbers of children with successful and interpretable lung function test data (spirometry test) at recruitment were 187 (HIV negative), 192 (HIV positive on ART), and 153 (HIV positive/ART-naive).

^d^CD4 count was not measured for the uninfected group, and the data were available for 357/402 HIV-positive participants.

CMV DNAemia was detected in 8.9% of uninfected children, 24.0% of HIV-positive participants established on ART, and 38.4% of the HIV-positive ART-naive children. At baseline, the prevalence of clinically relevant CMV DNAemia (CMV VL ≥1000 copies/mL) in the uninfected, HIV-positive on ART, and HIV-positive ART-naive groups was 5.8%, 14.7%, and 22.6%, respectively (χ^2^ = 23.8, *P* < .001; Table 1).

### Association Between CD4 Count and CMV Viral Load

Of the 55 HIV-positive participants with clinically significant CMV DNAemia at enrollment, 52% were on ART for >2 years before joining the cohort. The proportion of children with clinically relevant CMV viremia was inversely associated with CD4 count at recruitment in the ART-stable group (Table 1). In a multivariate analysis controlling for repeated measures and adjusting for ART status, age, and sex, CD4 count <350 cells/µL was associated with more than twice the odds of CMV VL >1000 copies/mL (Table 2). After adjusting for CD4 count, age, and sex, no longer being on ART showed significant protection against high CMV DNAemia; however, each additional year on ART was associated with 0.84 the odds of CMV VL ≥1000 copies among ART-treated participants (Table 3), even after adjusting for CD4 count. HIV RNA <1000 copies/mL was not associated with CMV DNAemia among participants on ART (Table 3).

**Table 2.** Multivariate Model of Risk Factors for High Cytomegalovirus DNAemia in All Human Immunodeficiency Virus–positive Participants

Characteristic	Category	Univariate^a^		Multivariate^a^	
		OR (95% CI)	*P* Value	OR (95% CI)	*P* Value
CD4 count (cells/µL)	≥350	1	…	1	…
	<350	2.70 (1.32, 5.51)	.006	2.64 (1.25, 5.59)	.011
Gender	Male	1	…	1	…
	Female	0.85 (0.48, 1.49)	.563	0.86 (0.46, 1.58)	.618
Age (years; ref: 6 years)		0.96 (0.87, 1.06)	.425	0.94 (0.83, 1.06)	.313
ART status	Naive	1		1	…
	>6 months on ART	0.43 (0.24, 0.78)	.005	0.61 (0.32, 1.16)	.132

Abbreviations: ART, antiretroviral therapy; CI, confidence interval; OR, odds ratio; ref, reference group, controlling for clustering by individual.

^a^A total of 402 participants contributing 665 records were used in univariate analysis, while 367 participants contributing 591 records were used in model 1.

### Stunting Is Associated With HIV and High CMV DNAemia

At baseline, 29.2% of HIV-infected ART-naive participants and 34.3% of those on ART were stunted compared to 9.6% of HIV-uninfected participants (Table 1). In ART-naive HIV-infected participants, the prevalence of CMV VL ≥1000 copies/mL was higher among stunted than nonstunted participants (40.8% vs 14.3%, χ^2^ = 14.2, *P* < .001). In participants who were on ART or HIV negative, the prevalence of CMV VL ≥1000 copies/mL was similar among stunted and nonstunted participants (Table 1).

### Association of Lung Function With High CMV DNAemia

A total of 89 participants did not have spirometry measurements of sufficient quality to allow lung function assessment. Both reduced FVC and spirometric features of obstruction were more common in HIV-infected than HIV-uninfected participants, particularly obstruction, which was more prevalent among ART-naive participants (Table 1). At baseline, 29.1% of stunted children compared to 20.8% of nonstunted children had abnormal lung function (reduced FVC or obstruction) in all 3 groups combined (χ^2^ = 3.85, *P* = .05). Only in HIV-positive ART-naive participants was high CMV DNAemia prevalence greater among those with abnormal lung function (Table 1).

In a longitudinal analysis of ART-naive HIV-positive participants, reduced FVC was independently associated with high CMV DNAemia after adjusting for gender, age, CD4 count, and individual-level clustering (Table 4). Stunting and obstructive spirometry were not associated with CMV VL. Similar analysis in HIV-positive participants on ART revealed that CD4 count <350 cells/µL and stunting (albeit weakly) were independently associated with high CMV DNAemia, while longer time on ART was associated with lower odds of high CMV DNAemia (Table 3). Abnormal lung function was not included in the model as there was no evidence it was associated with outcomes of participants on ART in univariate analysis.

**Table 3.** Multivariate Model of Risk Factors for High Cytomegalovirus DNAemia in Participants on Antiretroviral Therapy

Characteristic	Category	Univariate^a^		Multivariate: *A Priori* Only^a^		Multivariate: *A Priori* or Associated With Outcome^a^	
		OR (95% CI)	*P* Value	OR (95% CI)	*P* Value	OR (95% CI)	*P* Value
Years since antiretroviral therapy initiation (ref: 0)	…	0.81 (0.69, 0.95)	.010	0.83 (0.69, 0.99)	.035	0.84 (0.70, 1.00)	.050
CD4 count (cells/µL)	≥350	1	…	1	…	1	…
	<350	3.92 (1.20, 12.77)	.023	3.75 (1.09, 12.94)	.036	4.39 (1.20, 16.04)	.025
Gender	Male	1	…	1	…	1	…
	Female	0.64 (0.27, 1.51)	.311	0.69 (0.27, 1.74)	.430	0.80 (0.31, 2.10)	.655
Age (y; ref: 6 y)	…	0.99 (0.85, 1.15)	.884	0.94 (0.79, 1.12)	.469	0.91 (0.75, 1.09)	.305
Height for age	Normal	1	…	…	…	1	…
	Stunted	3.01 (1.17, 7.77)	.022	…	…	2.79 (0.97, 8.02)	.057
Lung function	Normal	1					
	Reduced forced vital capacity	1.22 (0.39, 3.80)	.727	…	…		
	Obstructed	0.41 (0.03, 6.52)	.527	…	…		
HIV RNA (337 records)	≥1000	1.01 (0.25, 4.03)	.988	…	…		
	<1000	1	…	…	…		

Abbreviations: CI, confidence interval; OR, odds ratio; ref, reference group, controlling for clustering by individual.

^a^A total of 262 participants contributing 434 records were used in univariate analysis; 256 participants contributing 411 records were used in model 1, and 251 participants contributing 405 records were used in model 2.

**Table 4.** Multivariate Model of Risk Factors for High Cytomegalovirus DNAemia in Antiretroviral Therapy–naive Human Immunodeficiency Virus–positive Participants

Characteristic	Category	Univariate^a^		Multivariate: *A priori* Only^a^		Multivariate: *A priori* or Associated With Outcome^a^	
		OR (95% CI)	*P* Value	OR (95% CI)	*P* Value	OR (95% CI)	*P* Value
CD4 count (cells/µL)	≥350	1	…	1	…	1	…
	<350	1.47 (0.67, 3.24)	.340	1.55 (0.68, 3.54)	.297	1.05 (0.40, 2.78)	.916
Gender	Male	1	...	1	...	1	...
	Female	1.10 (0.56, 2.16)	.793	1.15 (0.54, 2.45)	.714	1.17 (0.49, 2.79)	.723
Age (y; ref: 6 y)		0.97 (0.85, 1.10)	.619	0.95 (0.81, 1.11)	.514	0.94 (0.78, 1.14)	.552
Height for age	Normal	1	...	...	...	1	...
	Stunted	4.23 (1.47, 12.16)	.008	...	...	2.02 (0.78, 5.27)	.150
Lung function	Normal	1	...	...	...	1	...
	Reduced forced vital capacity	2.11 (0.89, 4.98)	.088	...	...	3.23 (1.23, 8.46)	.017
	Obstructed	1.47 (0.49, 4.41)	.485	...	…	2.04 (0.48, 8.70)	.336

Abbreviations: CI, confidence interval; OR, odds ratio; ref, reference group, controlling for clustering by individual.

^a^A total of 177 participants contributing 231 records were used in univariate analysis, 143 participants contributing 180 records were used in model 1, and 138 participants contributing 169 records were used in model 2.

When the outcome was defined as any detectable CMV, lung function and stunting were not associated with CMV DNAemia in multivariate analysis among ART-naive or ART-treated participants. Being on ART for at least 6 months was associated with lower odds of detectable CMV DNA in plasma (adjusted odds ratio, 0.54; 95% confidence interval, 0.31–0.93; *P* = .026; Supplementary Table 1).

Analysis of longitudinal samples taken 18 months apart from a subset of 159 children and adolescents with PHIV showed that 15.7% (20/127) of participants with undetectable CMV DNA at baseline had detectable levels of CMV DNA in their 18 month samples and vice versa (Table 5).

**Table 5.** Frequency of Cytomegalovirus DNAemia in a Subset of Longitudinal Samples From the Human Immunodeficiency Virus–positive Group

		CMV DNA at 18 Months		
		Detected	Not Detected	Total^a^
CMV DNA at baseline	Detected	11	21	32
	Not detected	20	107	127
	Total	31	128	159

Abbreviation: CMV, cytomegalovirus.

^a^Number of participants with 2 samples taken 18 months apart. The presence and quantity of CMV DNA was measured in both samples (baseline and follow-up) at the same time in a single plate to minimize intra- and interassay variability.

## DISCUSSION

We investigated the hypothesis that poorly controlled CMV coinfection, acquired in infancy in most African children, could contribute to the comorbidities of older children and adolescents with PHIV. We found a surprisingly high prevalence of CMV DNAemia in older children with PHIV, both those with a new HIV diagnosis who had not yet started ART and those regarded as stable on ART. CMV DNAemia >1000 copies/mL was significantly associated with 2 major PHIV comorbidities, stunting and reduced lung function.

CLD in older children with PHIV was first described in Zimbabwe and subsequently reported in Malawi [18] and Kenya [19]. Radiological features strongly suggest obliterative bronchiolitis (OB) as the main cause [7], in which inflammation of the small airway leads ultimately to partial or complete bronchiolar obstruction. In high-income settings, OB is most often associated with allograft immune responses following lung or hematopoietic stem cell transplantation [20]; subclinical CMV infection has been linked with increased OB risk after lung transplantation [21]. Reports from the southern hemisphere describe OB following severe respiratory infections in early childhood [22]. As the epidemiology of CMV infection is distinct between sub-Saharan Africa and developed countries, we postulated that early CMV infection could contribute to the striking comorbidities associated with PHIV in Africa [4].

The trajectory of CMV viremia and shedding after primary infection remains poorly understood. Children infected in infancy were reported to secrete CMV in urine until the age of 5 years. However, a large cross-sectional survey in the United States showed that 9.7% of the CMV immunoglobulin (Ig) G-positive population aged 6–49 years were excreting CMV in urine [23]. Urinary CMV shedding was most common in the youngest participants (26.6% in children aged 6–11 years) but was also detected in adolescents (6.5% in those aged 12–19 years). Even less is known about the dynamics of CMV viremia. In one study of hospitalized children aged 3 weeks to 2 years in Lusaka, Zambia, CMV was detected in the blood of 41% and was associated with being HIV infected, underweight, and presenting with meningitis [24]. Two age peaks of CMV DNAemia were noted, particularly in HIV-infected children, one in the first year of life and a second around 18–20 months of age. This was interpreted as either CMV reactivation or potentially reinfection. CMV DNAemia is rare in healthy adults (CMV DNA was found in only 2/416 CMV IgG-positive blood donors in the United States [25]). However, CMV is frequently reactivated during pregnancy and lactation, particularly in HIV-infected women, leading to viral shedding in the cervix and breastmilk. In HIV-uninfected pregnant women, CMV DNA is rarely detected in blood but may be found in the cervix of 37% CMV IgG-positive women, particularly during the third trimester [26]. In HIV-infected Kenyan women taking ART to prevent mother-to-child HIV transmission, CMV was detected in the cervix and breastmilk of 66% and 99% of participants, respectively, although CMV was only found in blood in 4.8% [27]. Increased CMV shedding in the cervix and breastmilk of HIV-infected women who are close to delivery increases the risk of both congenital and early CMV infection in their infants [27]. Although ART used to prevent mother-to-child HIV transmission during pregnancy reduces the risk of congenital CMV infection [13], the reduction in early postnatal CMV transmission is only modest [28].

The burden of severe opportunistic CMV infection, causing common AIDS-defining conditions such as retinitis, colitis, and esophagitis, has decreased substantially with the availability of ART. Nevertheless, studies of HIV-infected adults in the developed world have shown that subclinical CMV DNAemia (ie, CMV DNA detected in blood without clinical evidence of CMV disease) strongly predicts accelerated disease progression and early mortality, even in patients taking ART [29]. Increased levels of CMV antibodies or T-cell responses (potentially driven by CMV reactivation) are associated with both elevated immune activation [30] and the non-AIDS–defining comorbidities increasingly seen in the aging population of HIV-infected people on long-term ART [31]. There are fewer studies in Africa, but one study of South African miners with relatively early HIV disease in the pre-ART era reported that CMV DNAemia (5.2% prevalence) was associated with a 3-fold increase in mortality [32]. In that study, higher CMV VLs (≥1000 copies/mL, our cutoff for clinically significant CMV DNAemia) were linked with significantly higher mortality (adjusted hazard ratio, 3.65) [32].

A recent metaanalysis showed that early CMV coinfection led to accelerated disease progression in ART-untreated infants with PHIV [13]. However, there is little information about the impact of CMV coinfection on HIV-infected children taking cART, particularly in Africa. Maternal CMV DNAemia close to delivery was associated with increased mortality of both mother and baby in a Kenyan cohort with limited ART access [33]. HIV and CMV plasma VLs closely correlated in the HIV-CMV–coinfected infants in this study, thought to reflect CMV-driven immune activation that fueled HIV replication in activated T cells [34, 35]. As CMV replication (like HIV) is enhanced by inflammation, the virus has developed strategies to promote immune activation, including direct upregulation of proinflammatory cytokines such as interleukin (IL)-1β, tumor necrosis factor-α, and IL-6 [36]. The association of CMV infection with inflammation is more marked in HIV-infected patients [37]. CMV was recently shown to be replicating in the gut mucosa of HIV-infected adults (even those on ART); this was associated with disruption of mucosal integrity, translocation of gut microbial products, and increased inflammation [38]. A trial of the anti-CMV drug valganciclovir in HIV-infected adults with incomplete immune reconstitution on ART showed that CMV suppression was significantly associated with reduced T-cell activation [39].

Our study has strengths and some limitations. We studied a large well-characterized group of HIV-infected older children with PHIV, both treated and untreated, as well as age- and sex-matched controls from the same population. An important limitation is the lack of information about HIV RNAs in the ART-naive participants at enrollment (not routinely measured at the time in Zimbabwe.) The INHALE participants described as stable on ART may have elevated HIV RNAs because of poor adherence, which is characteristic of HIV-infected adolescents [40]. However, HIV RNA data, available from 337 of 434 samples from participants on ART, showed no association between HIV RNA and CMV DNAemia in univariate analysis. Last, our data do not allow us to determine the direction of associations, so we cannot conclude that CMV is a cause of stunting or CLD. An alternative interpretation would be that all 3 are independent but causally unrelated markers of progressive PHIV disease.

Additional studies are needed to establish the potential role of CMV reactivation in the pathogenesis of the serious complications of PHIV in Africa and to determine whether or not specific antiviral drugs such as valganciclovir might be useful in these conditions.

## Supplementary Data

Supplementary materials are available at *Clinical Infectious Diseases* online. Consisting of data provided by the authors to benefit the reader, the posted materials are not copyedited and are the sole responsibility of the authors, so questions or comments should be addressed to the corresponding author.

ciy961_suppl_Supplementary_Table

ciy961_suppl_Supplementary_Methods

## Notes

***Acknowledgments.*** The authors thank all the participants for their invaluable contributions to the study and the staff of the African Institute of Biomedical Science and Technology in Harare for their technical support.

***Financial support.*** This work was supported by the Wellcome Trust (095878/Z/11/Z), the Nina-Ireland Lung Development Programme, the John Fell Fund (Oxford University), the International AIDS Society Collaborative Initiative for Paediatric human immunodeficiency virus Education and Research programme (2016/345-YIN), and the Royal Society of Tropical Medicine and Hygiene. The funding sources had no role in the writing and submission of the work described above.

***Potential conflicts of interest.*** R. A. F. received grants from the Wellcome Trust during the course of the study. L. M. Y. received grants from the International AIDS Society-Collaborative Initiative for Paediatric HIV Education and Research programme and the Royal Society of Hygiene and Tropical Medicine during the course of this study. S. R. J. received grants from John Fell fund (University of Oxford), International AIDS Society, and the Royal Society of Hygiene and Tropical Medicine during the conduct of the study and grants from International Research Centre for Medical Sciences, University of Kumamoto in Japan, British HIV Association, Rosetrees Trust, Research Council of Norway, and Association of Physicians Developing Countries grant scheme outside the submitted work. All other authors: No reported conflicts. All authors have submitted the ICMJE Form for Disclosure of Potential Conflicts of Interest. Conflicts that the editors consider relevant to the content of the manuscript have been disclosed.
