# Supplementary material for: Unexpectedly High Prevalence of Cytomegalovirus DNAemia in Older Children and Adolescents With Perinatally Acquired Human Immunodeficiency Virus Infection
Source: Clin Infect Dis. 2019 Mar 4;69(4):580–7. doi: 10.1093/cid/ciy961 (PMC6669294; doi:10.1093/cid/ciy961)
Supplement: ciy961_suppl_Supplementary_Table [file ciy961_suppl_supplementary_table.docx]

Supplementary Table 1: Multivariate model of risk factors for any detectable CMV DNA-aemia in all HIV positive participants

|  | Category | Univariate^a^ | | Multivariate^a^ | |
| --- | --- | --- | --- | --- | --- |
|  |  | OR (95% CI) | p-value | OR (95% CI) | p-value |
| CD4 count (cells/µl) | 350+ | 1 | - | 1 | - |
|  | <350 | 3.86 (2.02, 7.40) | <0.001 | 3.72 (1.91, 7.26) | <0.001 |
| Gender | Male | 1 | - | 1 | - |
|  | Female | 0.87 (0.52, 1.44) | 0.578 | 0.91 (0.54, 1.52) | 0.712 |
| Age in years (ref: 6) |  | 0.94 (0.86, 1.03) | 0.175 | 0.92 (0.83, 1.02) | 0.107 |
| ART status | Naïve | 1 |  | 1 | - |
|  | >6 months on ART | 0.39 (0.23, 0.65) | <0.001 | 0.54 (0.31, 0.93) | 0.026 |

a: 402 participants contributing 665 records were used in univariate analysis while 367 participants contributing 591 records were used in model 1; ref: reference group. Controlling for clustering by individual
