# Supplementary material for: Unexpectedly High Prevalence of Cytomegalovirus DNAemia in Older Children and Adolescents With Perinatally Acquired Human Immunodeficiency Virus Infection
Source: Clin Infect Dis. 2019 Mar 4;69(4):580–7. doi: 10.1093/cid/ciy961 (PMC6669294; doi:10.1093/cid/ciy961)
Supplement: ciy961_suppl_Supplementary_Methods [file ciy961_suppl_supplementary_methods.docx]

**Supplementary methods**

*CMV detection and quantification*

Kits were purchased as a single lot and were used following the manufacturer’s instructions. Each qPCR reaction contained a heterologous internal control to validate PCR efficiency. Each patient sample was tested in duplicates to minimise false negative results and any sample with discordant results was repeated. To minimise other sources of variation, all CMV DNA measurements were performed in the same laboratory using the same instrument by the same person (L-MY). Each experimental batch contained 4 quantification standards as positive controls, with known CMV DNA concentrations ranging from 10 -10^4^ IU/µl, which were used to generate standard curves. The analytical sensitivity (Limit of Detection - LoD) of the RealStar CMV kit is defined as the concentration of CMV DNA molecules that can be detected with a positivity rate of 95%. A dilution series of CMV DNA was analysed in eight replicates. Using the World Health Organisation (WHO) International Standard for Human Cytomegalovirus and the Applied Biosystems real-time PCR instruments the LoD was 0.668 IU/μl (CI ≥ 95%: 0.323 – 2.258 IU/μl). The conversion factor for DNA values to copies/mL was 0.6.

Of note, longitudinal samples (baseline and follow-up) were tested in the same plate, at the same time, with the same lot of reagents, by the same operator using the same q-PCR instrument as described above.

*Statistical analysis*

We described the characteristics of the participants at baseline. Data collected in the first 6 months after ART initiation were excluded from analysis. We included all other records where blood was taken and CMV DNA-aemia was measured. Repeat measures were coded into 6-month intervals. For multivariate analysis of time-varying outcomes we used longitudinal mixed-methods logistic regression, with a random effect to account for repeated measures of the same participants and fixed effects for age and gender. ART naïve participants and those stable on ART were modelled separately. Data were analysed using Stata v14.1 (StatCorp, Texas, USA). Variables that changed over time were CMV DNA-aemia, CD4 count, HIV VL, age and time since ART initiation. Variables measured only at baseline were stunting, lung function and gender.
